# Metastatic Secondary Fibrosarcoma of Bone Responsive to Repeated Courses of Ifosfamide and Associated With Hypoglycemia

**DOI:** 10.1080/13577140310001607329

**Published:** 2003-06

**Authors:** Elaine S. Bouttell, N. Wilson Rodger, Vivien H. C. Bramwell

**Affiliations:** 1 Department of Medical Oncology London Regional Cancer Centre 399 Royal Avenue Kelowna, BC London V1Y 5L3 Canada; 2 Division of Endocrinology Department of Medicine St. Joseph's Hospital Ontario London Canada

## Abstract

We present a case of a 40-year-old man with secondary fibrosarcoma of bone, arising from a non-ossifying fibroma. He subsequently developed metastatic disease that responded to four successive chemotherapy courses, the last three using the same dose/schedule of single agent ifosfamide. Eventual rapid progression of a huge intra-abdominal mass was associated with the syndrome of extrapancreatic tumour hypoglycemia (EPTH). The clinicopathological behaviour of fibrosarcoma of bone, and the mechanism of EPTH are discussed.


*Sarcoma*, June 2003, *VOL. 7, NO. 2, 81–85*

Taylor & Francis healthsciences

CASE REPORT

### Metastatic secondary fibrosarcoma of bone responsive to repeated courses of ifosfamide and associated with hypoglycemia

ELAINE S. BOUTTELL[1], N. WILSON RODGER[2] & VIVIEN H.C. BRAMWELL[1]

[1]*Department of Medical Oncology, London Regional Cancer Centre &* [2]*Division of Endocrinology, Department of Medicine, St. Joseph's Hospital, London, Ontario, Canada*

**Abstract**

We present a case of a 40-year-old man with secondary fibrosarcoma of bone, arising from a non-ossifying fibroma. He subsequently developed metastatic disease that responded to four successive chemotherapy courses, the last three using the same dose/schedule of single agent ifosfamide. Eventual rapid progression of a huge intra-abdominal mass was associated with the syndrome of extrapancreatic tumour hypoglycemia (EPTH). The clinicopathological behaviour of fibrosarcoma of bone, and the mechanism of EPTH are discussed.

**Key words:** *fibrosarcoma, hypoglycemia, IGF-II*

### Introduction

Fibrosarcoma arising from the medullary, or less commonly, from the periosteal region of bone, represents only 5% of the primary malignant tumours of bone.[1–3] These lesions may also develop secondary to previous radiation, or arise from pre-existing benign conditions such as fibrous dysplasia, Paget's disease, bone infarct or cyst, osteomyelitis, and giant-cell tumour of bone.[1–3] Fibrosarcoma of bone has the same histological features as soft tissue fibrosarcoma, including spindle shaped tumour cells and interlacing bundles of collagen fibres without any other type of histological differentiation such as cartilage or bone formation.[1–4]

The syndrome of extrapancreatic tumour hypoglycemia (EPTH) was described in 1930, in a patient with hypoglycemia in association with a large fibrosarcoma.[5] This syndrome is well recognized in patients with sarcoma, as well as other non-islet cell tumours of mesodermal or epithelial origin.[6] Such tumours may be benign or malignant, but are often slow growing and very large.[1] The mechanism is felt to be related to the production of insulin-like growth factor-II (IGF-II) by the tumour.[7]

We present an unusual case of a man with fibrosarcoma showing prolonged and repeated responses to treatment with single agent ifosfamide for metastatic disease. Toward the end of his life, rapid tumour progression with the development of massive intra-abdominal disease was associated with severe hypoglycemia requiring treatment with a glucagon infusion to maintain his blood glucose level in the normal range.

### Case report

A 40-year-old man required open reduction internal fixation for a pathological fracture of the distal right femur after a fall in October 1994. The pathology was consistent with a benign non-ossifying fibroma. In February 1995, the patient noted right leg pain and edema, and surgery was scheduled for removal of a compression screw from the supracondylar fixation device. At surgery, tumour was found in the surrounding soft tissue. The pathology revealed evolution of the lesion to a low grade fibrosarcoma of bone extending into the surrounding soft tissue.

The patient was evaluated at the London Regional Cancer Centre in June of 1995. At that time, there was a firm non-tender mass in the right thigh with wasting of the quadriceps and gastrocnemius. A radiograph of the right femur showed the previous pathological fracture site, side plate and screws with mottled bone density, lytic lesions and sclerosis at the distal end. A periosteal mass extending into soft tissues was present. Magnetic resonance imaging (MRI) of the right leg revealed a mass in the right biceps femora measuring $12 \times 10 \times 12.5$ cm and a

Correspondence to: Elaine S. Bouttell, MD, Cancer Centre for the Southern Interior, 399 Royal Avenue, Kelowna, BC, Canada V1Y 5L3. Tel: +1-250-979-6623; Fax: +1-250-712-3911.

ISSN 1357-714X print/ISSN 1369-1643 © 2003 Taylor & Francis Ltd
DOI: 10.1080/13577140310001607329

second mass, 5 cm proximal to this, in the vastas intermedius measuring 3.8 × 3.8 × 3.5 cm. A bone scan showed increased tracer uptake throughout the distal 3/4 of the right femur. Although increased uptake was also present in the right 8th and 9th ribs anteriorly, and a left rib posteriorly, radiographs of the ribs were normal. Chest radiograph (CXR) showed multiple calcified granulomata, unchanged from October 1994. The benign appearance of these lesions was confirmed on computed tomography (CT) scan of the thorax. A right above-knee amputation was performed in August 1995. Pathology review by an experienced sarcoma pathologist described a fibrosarcoma grade II/IV, with more aggressive histological features than seen in the bone tumour initially resected in October 1994. The resection margins were clear and no vascular invasion was identified. Adjuvant treatment was not given.

The patient was well until August 1996 when a fall resulted in a pathological fracture of the surgical neck of the right humerus. CXR showed multiple new bilateral pulmonary nodules as well as the previously noted calcified granulomata. Pulmonary metastases were confirmed on CT scan of the thorax. A bone scan revealed increased tracer uptake in the intertrochanteric region of the right femur, 7th left rib anteriorly, proximal right humerus and humeral head, and in the left femur from the intertrochanteric region to the mid-shaft. MRI of the pelvis showed a central pelvic soft tissue mass measuring 10 × 11 cm, and a smaller 1 × 1.5-cm mass in the right ischium. The patient was treated with palliative radiotherapy (20 Gy in five fractions) to the right shoulder but soon after developed symptomatic epidural cord compression at T5, and received palliative radiotherapy from T3 to T7, with symptomatic improvement. He then received seven cycles of doxorubicin 75 mg/m$^2$ intravenously (i.v.) every 3 weeks from October 1996 to March 1997, at which time the total cumulative dose of doxorubicin given was 525 mg/m$^2$. He achieved a partial response with a 50% decrease in his pulmonary metastases and significant shrinkage of the pelvic masses.

He remained well off treatment until August 1997 when he developed significant neck pain and received palliative radiotherapy to a left paraspinal soft tissue mass at the C3 level. There was progression of lung and intra-abdominal/pelvic metastases and in September 1997 he began treatment with ifosfamide 5 g/m$^2$ 24-h infusion given with hydration and Mesna every 3 weeks. He completed 12 cycles in May 1998, again achieving a partial response in the pulmonary metastases, as well as shrinkage of the intra-abdominal/pelvic masses. He was subsequently followed off treatment.

In November 1998, the patient presented with a new soft tissue mass in the right anterior chest wall and progression of pulmonary metastases. It had been 6 months since completion of his first course of ifosfamide, and treatment was re-instituted using the same regimen. Twelve more cycles of ifosfamide were completed in August 1999, with partial response, and he was again followed off treatment.

In January 2000, the patient developed chest wall pain secondary to recurrence of the right anterior chest wall mass. There was also progression of the pulmonary metastases on CXR. A third course of ifosfamide was instituted. In October 2000, he had completed 11 more cycles, with a further partial response of the chest wall mass and pulmonary metastases. However, the following month he presented with new left lower abdominal pain and a palpable pelvic mass. A CT scan revealed multiple intra-abdominal soft tissue masses although no liver metastases, lytic lesions of the right hemipelvis and proximal femur, and a soft tissue mass in the right upper thigh. The lung metastases had not progressed. A biopsy of the left pelvic mass was arranged to rule out a second primary malignancy due to the unusual development of increasing abdominal masses without progression of pulmonary metastases. The pathology was consistent with myxoid spindle cell sarcoma.

On the day of the biopsy, the patient became pale and diaphoretic and experienced a syncopal episode. His blood glucose was 1.5 mmol/l (normal range 3.4–11.0 mmol/l). He received intravenous dextrose and was admitted to hospital. There was no evidence of infection. He was started on a diet of frequent meals (q4h) to avoid recurrent hypoglycemia. Investigations revealed normal liver function, an appropriately low insulin level of < 2.0 mU/l (normal < 17.0 mU/l), and low C-peptide level of 61 pmol/l (normal range 166–990 pmol/l), with an appropriately elevated serum cortisol level of 968 nmol/l (normal range 119–618 nmol/l). Insulin-like growth factor I (IGF-I) level was normal at 137 μg/l (normal range 75–306 μg/l). Insulin-like growth factor II (IGF -II) level was measured using a two-site immunoradiometric assay (Diagnostic Systems Laboratories Inc., Webster, TX). It was found to be normal at 699 ng/ml (normal range 519–1067 ng/ml) and was 766 ng/ml on repeat testing. Total parenteral nutrition to supplement his oral intake, as well as intermittent boluses of intravenous dextrose were required to maintain his blood glucose level over 3.0 mmol/l. Later, the addition of a glucagon infusion was implemented to maintain his blood glucose in the normal range. In an attempt to control the disease, treatment with dacarbazine 1000 mg/m$^2$ was initiated. However, his abdominal mass continued to progress rapidly and he died 2 weeks later.

### Discussion

Primary fibrosarcoma of bone is recognised as an entity distinct from soft tissue fibrosarcoma and tends to carry a poorer prognosis.[2,3] However, these neoplasms share pathological features of spindle-shaped tumour cells with interlacing bundles of collagen fibres without osseous or cartilagenous differentiation.[1–4] In Broder's grading system, well and moderately differentiated tumours correspond to grades I and II, respectively, while grades III and IV refer to high grade, poorly differentiated tumours.[4] Fibrosarcoma of bone exhibits a predilection for long tubular bones, especially around the knee in the distal femur and proximal tibia. However, fibrosarcomas can also occur in the humerus, as well as in the bones of the axial skeleton.[2–4] Patients are usually in their third to sixth decade of life, most commonly present with pain, and up to one-third may develop a pathological fracture.[2] An associated soft tissue mass is present in 85% of cases.[2] The radiographic appearance of high grade lesions is often a patchy lytic 'moth-eaten' pattern which is poorly marginated.[1–4]

The 5-year overall survival rate for primary fibrosarcoma of bone is 34–40%, although lesions that are limited to bone carry a better prognosis (64% 10-year overall survival) compared to those with soft tissue invasion (13% 10-year overall survival).[2,4] Limb-salvage procedures result in the same overall survival rate as amputations as long as wide resection margins are achieved.[4] Adjuvant radiation therapy may reduce the high risk of local recurrence following excision with positive margins; however, overall survival remains poor in this setting.[3] Adjuvant chemotherapy has an established role in osteosarcomas and may have similar efficacy in malignant fibrous histiocytoma of bone (MFH-B).[8,9] However, its role in the rare entity of fibrosarcoma of bone is unknown. For soft tissue sarcomas (STS), adjuvant doxorubicin and/or ifosfamide may also have benefits for large, high grade tumours, especially of the extremities, but this is more controversial.[10]

Multivariate analyses of clinicopathological series of primary fibrosarcoma of bone, have shown that age greater than 40 years, tumour in the axial skeleton, high tumour grade, and positive resection margins are all risk factors for disease recurrence and lower overall survival.[2,3] Fibrosarcoma spreads hematogenously, with the most frequent sites of metastases being lung and bone.[1–3] Pulmonary metastases represent the leading cause of death in sarcoma. Complete resection of metastatic sites in unselected soft tissue and bone sarcomas may result in an overall 5-year survival rate of 15–38%.[2,11] However, as in our patient's case, unresectable multiple or bilateral pulmonary metastases are common.

Many studies have shown that metastases to lung and soft tissue are more responsive to chemotherapy than metastatic disease in liver and bone. In metastatic STS, the most active single agents include doxorubicin, ifosfamide, and dacarbazine, with reported response rates of 26, 18–21 and 16%, respectively.[12] Definitive evidence favouring combination chemotherapy over the use of single agents is not available. Many combinations show an improvement in response rate at the expense of significantly increased toxicity without an increase in overall survival.[12–14] It is unknown whether fibrosarcomas originating in bone are more responsive to regimens active in bone sarcomas (osteosarcoma or MFH-B) or STS. Because of this uncertainty we chose to treat our patient with consecutive single agents (doxorubicin and ifosfamide) that have documented activity in both bone and soft tissue sarcomas. To our knowledge, there has not been a previous report of a patient with metastatic fibrosarcoma of bone who has shown such repeated and prolonged responses to treatment with single agent ifosfamide.

In a patient receiving chemotherapy for an intra-abdominal malignancy and presenting with hypoglycemia, serious treatable causes, such as abdominal sepsis, should be excluded. Chronic renal or liver disease, and less commonly, deficiency of counter-regulatory hormones should be considered. The mechanism of hypoglycemia associated with non-islet cell tumours has puzzled many since its description by Nadler and Wolfer in 1929, and by Doege in 1930.[5,15] Extrapancreatic tumour hypoglycemia (EPTH) is characterised by fasting hypoglycemia accompanied by symptoms of neuroglycopenia. Associated metabolic features are similar to those seen with increased insulin activity, including inhibition of hepatic glycogenolysis and gluconeogenesis, inhibition of lipolysis, and increased peripheral glucose consumption, partly by the tumour itself, but mainly due to increased glucose uptake by skeletal muscle.[16,17] These features occur with low circulating insulin levels and inhibition of the counter-regulatory mechanism of growth hormone secretion.[7,18] As a result, insulin-like growth factors capable of stimulating insulin receptors were felt to be the most likely candidates as the cause of EPTH. However, IGF-I levels are consistently suppressed in these patients and many early studies showed IGF-II levels were in the normal range when measured by radioreceptor or radioimmunoassays (RIA), such as the assay used for our patient.[18–21] There have been a number of hypotheses, but the definitive explanation for this is uncertain.

In 1988, Daughaday *et al.* reported a case of a patient with leiomyosarcoma associated with EPTH whose serum IGF-II levels were normal using radioreceptor assay and RIA. However, when serum was acidified and fractionated using a BioGel P-60 column, 70% of the total IGF-II eluted was in the high molecular weight range (big IGF-II), whereas most of the IGF-II in normal serum was of lower molecular weight. They tested tumour samples using this same method and showed that 77% of the total IGF-II consisted of the high molecular weight form.[7] Others have found that these tumours contain greater than 100 times the amount of IGF-II mRNA found in normal human liver or adipose tissue.[22,23] Both IGF-I and II are bound to specific high molecular weight carrier proteins in the circulation, with less than 2% found in the free form.[24] In normal serum, 70–80% of IGF-I and II are found within a 150-kDa complex which is a trimer consisting of IGF, IGF binding protein-3 (IGFBP-3), and an acid-labile glycoprotein (α-subunit).[18,24,25] Normal levels of both IGFBP-3 and the α-subunit are dependent on growth hormone secretion.[18] This large complex is sequestered in the vascular compartment by the capillary barrier, is not bioavailable to tissues, and thus has a long serum half-life of 12–16 h.[26] Normally, a smaller proportion (20–30%) of IGF-II is present in a smaller 50-kDa complex that can cross the capillary barrier and so is the main form that is bioavailable to tissues. This complex contains IGF bound mainly to IGFBP-2 and some IGFBP-3, and is more rapidly cleared from the circulation, with a half-life of 30 min.[21,26] In patients with EPTH, the 150-kDa complex may be reduced due to inhibition of growth hormone by IGF resulting in decreased synthesis of the IGFBP-3 and the α-subunit. The IGF-II content of the 50-kDa complex is then increased, which may account for its increased bioavailability to tissues despite normal levels of total IGF-II.[18,27]

Continuous intravenous glucagon infusion for the treatment of EPTH was first described by Samaan *et al.*[28] However, prior to that, intramuscular injection of zinc glucagon had been used in the treatment tumour-induced hypoglycemia.[16,29] Glucagon stimulates hepatic glucose production by glycogenolysis and gluconeogenesis. However, when hypoglycemia is due to liver metastases with hepatic failure, poor glycogen reserve may result in failure to respond to glucagon administration.[18] If normalisation of blood glucose levels is achieved, continuous glucagon infusion using a portable pump can be administered on an outpatient basis.[18] However, tumor eradication by surgery, radiotherapy or chemotherapy is crucial for long-term control of glucose levels. In our patient, the syndrome of EPTH was associated with a fifth metastatic relapse that occurred after responses to four prolonged courses of chemotherapy (three to the same drug). Emergence of the syndrome seemed to be associated with aggressive drug-resistant disease leading to the patient's early demise.

### Acknowledgements

Special thanks to Dr. David Hill, Dr. Lily Huang, Catherine Bond-Mills, BSc Pharm, and Cheryl Sigfrid, RD.

### References

1. Huvos AG, Higinbotham NL. Primary fibrosarcoma of bone. A clinicopathologic study of 130 patients. *Cancer* 1975; 35: 837–47.
2. Huvos AG. Fibrosarcoma of bone. In: Huvos AG, ed. *Bone Tumors: Diagnosis, Treatment and Prognosis*. 2nd edn. Philadelphia, PA: WB Saunders, 1991: 413–27.
3. Papagelopoulos PJ, Galanis E, Frassica FJ, *et al.* Primary fibrosarcoma of bone. Outcome after primary surgical treatment. *Clin Orthop Rel Res* 2000; 373: 88–103.
4. Scott SM, Reiman HM, Pritchard DJ, Ilstrup DM. Soft tissue fibrosarcoma. A clinicopathological study of 132 cases. *Cancer* 1989; 64: 925–31.
5. Doege KW. Fibrosarcoma of the mediastinum. *Ann Surg* 1930; 92: 955–60.
6. Turner RC. Hypoglycemia. In: Weatherall DJ, Ledingham JGG, Warrell DJ, eds. *Oxford Textbook of Medicine*. 3rd edn. Oxford: Oxford University Press, 1996: 1505–12.
7. Daughaday WH, Emanuele MA, Brooks MH, *et al.* Synthesis and secretion of insulin-like growth factor II by a leiomyosarcoma with associated hypoglycemia. *New Engl J Med* 1988; 319: 1434–40.
8. Bramwell VHC. The role of chemotherapy in the management of non-metastatic operable extremity osteosarcoma. *Semin Oncol* 1997; 24: 561–71.
9. Bramwell VHC, Steward WP, Nooji M, *et al.* Neoadjuvant chemotherapy with doxorubicin and cisplatin in malignant fibrous histiocytoma of bone: a European Osteosarcoma Intergroup study. *J Clin Oncol* 1999; 17: 3260–9.
10. Bramwell VHC. Editorial: Adjuvant chemotherapy for adult soft tissue sarcoma – is there a standard of care? *J Clin Oncol* 2001; 19: 1235–7.
11. van Geel AN, Pastorino U, Jaunch KW, *et al.* Surgical treatment of lung metastases: The European Organization for Research and Treatment of Cancer, Soft Tissue and Bone Sarcoma Group study of 255 patients. *Cancer* 1996; 77: 675–82.
12. Demetri GD, Elias AD. Results of single agent and combination chemotherapy for advanced soft tissue sarcomas. Implications for decision making in the clinic. *Hematol Oncol Clin N Am* 1995; 9: 765–85.
13. Bramwell VHC, Anderson D, Charette ML. Doxorubicin-based chemotherapy for the palliative treatment of adult patients with locally advanced or metastatic soft tissue sarcoma: a meta-analysis and clinical practice guideline. *Sarcoma* 2000; 4: 103–12.
14. Santoro A, Tursz T, Mouridsen H, *et al.* Doxorubicin versus CYVADIC versus doxorubicin plus ifosfamide in first-line treatment of advanced soft tissue sarcomas: A randomized study of the European Organization for Research and Treatment of Cancer, Soft Tissue and Bone Sarcoma Group. *J Clin Oncol* 1995; 13: 1537–45.
15. Nadler WH, Wolfer JA. Hepatogenic hypoglycemia associated with primary liver cell carcinoma. *Arch Intern Med* 1929; 44: 700–10.
16. Jakob A, Meyer UA, Flury R, *et al.* The pathogenesis of tumor hypoglycemia: Blocks of hepatic glucose releases and of adipose tissue lipolysis. *Diabetologia* 1967; 3: 506–14.
17. Moller N, Blum WF, Mengel A, *et al.* Basal and insulin stimulated substrate metabolism in tumor induced hypoglycemia. Diabetologia 1991; 34: 17–20.
18. Hoff AE, Vassilopoulou-Sellin R. The role of glucagon administration in the diagnosis and treatment of patients with tumor hypoglycemia. Cancer 1998; 82: 1585–92.
19. Daughaday WH, Trivedi B, Kapadia M. Measurement of insulin-like growth factor II by a specific radioreceptor assay in serum of normal individuals, patients with abnormal growth hormone secretion and patients with tumor associated hypoglycemia. *J Clin Endocrinol Metab* 1981; 53: 289–94.

20. Froesch ER, Zapf J, Widmer U. Hypoglycemia associated with non-islet cell tumor and insulin-like growth factors. *New Engl J Med* 1982; 306: 1178–9.
21. Widmer U, Zapf J, Froesch ER. Is extrapancreatic tumor hypoglycemia associated with increased levels of insulin-like growth factor II? *J Clin Endocrinol Metab* 1982; 55: 833–9.
22. Zapf J, Futo E, Peter M, Froesch ER. Can 'big' IGF-II in serum of tumor patients account for the development of extrapancreatic tumor hypoglycemia (EPTH)? *J Clin Invest* 1992; 990: 2574–84.
23. Lowe WL Jr, Roberts CT Jr, LeRoith D, *et al*. Insulin-like growth factor II in non-islet cell tumors associated with hypoglycemia: increased levels of messenger ribonucleic acid. *J Clin Endocrinol Metab* 1989; 69: 1153–9.
24. Zapf J. Role of insulin-like growth factor II and IGF binding proteins in extrapancreatic tumor hypoglycemia. Horm Res 1994; 42: 20–6.
25. Zapf J, Schmid Ch, Froesch ER. Biological and immunological properties of insulin-like growth factors (IGF) I and II. *Clin Endocrinol Metab* 1984; 13: 3–30.
26. Guler HP, Zapf J, Schmid C, Froesch ER. Insulin-like growth factors I and II in health men. Estimation of half-lives and production rates. *Acta Endocrinol* 1989; 121: 753–8.
27. Daughaday WH, Kapadia M. Significance of abnormal serum binding of insulin-like growth factor II in the development of hypoglycemia in patients with non-islet cell tumors. *Proc Natl Acad Sci USA* 1989; 86: 6778–82.
28. Samaan NA, Pham FK, Sellin RV, *et al*. Successful treatment of hypoglycemia using glucagon in a patients with an extrapancreatic tumor. *Ann Intern Med* 1990; 113: 404–6.
29. Roth H, Their S, Segal S. Zinc glucagon in the management of refractory hypoglycemia due to insulin-producing tumors. *New Engl J Med* 1966; 274: 493–7.
